# Alcohol and cardio-respiratory deaths in Chinese: a population-based case-control study of 32,462 older Hong Kong adults

**DOI:** 10.1186/1471-2458-9-49

**Published:** 2009-02-05

**Authors:** C Mary Schooling, Tai Hing Lam, Sai Yin Ho, Yao He, Kwok Hang Mak, Gabriel M Leung

**Affiliations:** 1 Department of Community Medicine and School of Public Health, The University of Hong Kong, 21 Sassoon Road, Pokfulam, Hong Kong, PR China; 2 Institute of Geriatrics, Chinese PLA General Hospital, Beijing, PR China; 3 Department of Health, Student Health Service, 4/F Lam Tin Polyclinic, Kowloon, Hong Kong, PR China

## Abstract

**Background:**

In observational studies moderate alcohol use reduces cardio-respiratory mortality. However observational studies may be biased by many factors including residual confounding by unmeasured differences between moderate alcohol users and other groups or by changes in alcohol use with ill-health and aging. We used two different analytic strategies in an under-studied population, i.e. southern Chinese, to provide an assessment of the specific impact of moderate alcohol use on mortality from ischemic heart disease (IHD) and chronic obstructive pulmonary diseases (COPD).

**Methods:**

In a population-based case-control study of all adult deaths in Hong Kong Chinese in 1998, we used adjusted logistic regression to compare alcohol use in decedents aged ≥ 60 years from IHD (2270) and COPD (1441) with 10,320 living and 9043 dead controls (all non-alcohol related deaths). We also examined whether the association of alcohol use with death from IHD or COPD varied with sex or smoking status.

**Results:**

Using living controls and adjusted for age, socio-economic status and lifestyle, occasional and moderate alcohol use were generally associated with lower mortality from IHD and COPD. However, using dead controls the protection of occasional and moderate alcohol use appeared to be limited to ever-smokers for IHD (odds ratio (OR) 0.58, 95% confidence interval (CI) 0.46 to 0.73 for moderate compared to never-use in ever-smokers, but OR 1.07, 95% CI 0.76 to 1.50 in never-smokers), and possibly to men for COPD. High alcohol use was associated with lower IHD mortality and possibly with lower COPD mortality.

**Conclusion:**

High levels of alcohol use in an older Chinese population were associated with lower IHD mortality. Moderate alcohol use was less consistently protective against IHD mortality. Alcohol use was associated with lower COPD mortality particularly in men, either due to some yet to be clarified properties of alcohol or as the artefactual result of genetic selection into alcohol use in a Chinese population. Given the increasing use of alcohol in China with economic development, other designs and analytic strategies are needed to assess the impact of alcohol in this population, so that an evidence-based public health policy can be formulated.

## Background

Many observational studies have shown that moderate alcohol use is associated with lower mortality from ischemic heart disease (IHD) [1] and ischemic stroke [2], but not hemorrhagic stroke [2]. Alcohol is also associated with lower respiratory mortality [3]. Despite the weight of evidence, there are counter examples [4,5], and a lack of good biological reasons for the negative association between alcohol use and respiratory mortality. Observational studies, prospective or case-control, cannot distinguish whether the protection offered by alcohol is causal or due to moderate alcohol use acting as a marker for other mortality related factors, such as healthier behaviors [6,7], more social integration [8] and better health status [9]. Self-selection of prudent or healthy moderate alcohol users might be evident as non-specific or systematic benefits, similar to that seen for hormone replacement therapy (HRT) in the observational studies now realized to be over-stated by selection into HRT use [10]. There are three prospective studies concerning alcohol use and cause specific cardiovascular mortality from China [11-13]. One found moderate alcohol use significantly protective against IHD, and non-significantly protective against liver cancer, lung cancer, hepatic cirrhosis and injuries and accidents with similar effect sizes but fewer deaths from the later causes [11]. The other two studies had few IHD deaths; one was too small to examine IHD separately [12], and the other found no association between moderate alcohol use and IHD mortality [13].

Prospective observational studies do not offer any obvious solution to this self-selection bias. Mendelian randomization studies provide a way forward, but few have been carried out concerning the effects of alcohol, and none on mortality. Meanwhile other less popular study designs, especially in non-western populations, may offer unique insights. Mortality case-control studies are rarely used because of the difficulty of selecting appropriate controls. Nevertheless in such a study the odds ratio obtained using dead controls (e.g. other deaths) produces an unbiased estimator of the mortality rate ratio (or relative risk) if the exposure in question has no effect on relative risk in these other deaths [14]. Moreover, such a comparison may reduce systematic self-selection of healthy moderate alcohol users, because a comparison with other deaths automatically compares people with a similar proximity to death and thereby a broadly similar state of health. In addition, recall bias by cause of death is unlikely. Causes of death positively associated with alcohol use are reasonably well-defined [15], so using selected dead controls in a mortality case-control study provides an opportunity to validate whether alcohol has a specific protective effect for some relevant cardiovascular and respiratory diseases.

We have previously used a population based case-control study of all deaths of ethnic Chinese in Hong Kong in 1998 and similar living controls to evaluate exposures potentially associated with mortality, such as smoking [16]. Here, we similarly examine the potential protective effect of moderate alcohol use on IHD and chronic obstructive pulmonary disease (COPD) mortality using living controls; we also used dead controls to provide a potentially less confounded assessment of the specific impact of moderate alcohol use on mortality from IHD and COPD in an under-studied and potentially different population.

## Methods

### Sources of data

The LIMOR (LIfestyle and MORtality) study is a population-based case control study of adult deaths among ethnic Chinese residents in Hong Kong during mid-December 1997 to mid-January 1999. The study was originally designed to examine the effect of smoking on mortality [16]. It captured 81% of all deaths registered during the study period. Information on demographic characteristics and health behaviors ten years previously of the decedent (the case) was collected from the person registering the death (the informant), who was usually one of the more educated members of the decedent's family. The same information was also obtained from the informant for a living person (a living control), who was someone other than the informant – either the decedent's spouse or a person, preferably aged 60 or above, whose habits ten years ago the informant was also familiar with. These were the only selection criteria given to the informant and no information was collected on the medical history of the controls, as it would undoubtedly have been incomplete. Information was always obtained from a third-party, who was usually an adult child of the participant, often living in close, multi-generational Chinese families. Information on health behaviors ten years previously was specifically sought to avoid reverse causality induced by changes due to ill-health. Proxy reports were chosen firstly for expediency, secondly because Chinese society with its collectivist values and non-phonetic language promotes potentially better memories focused more on others and thirdly because proxies have been shown to be capable of providing reliable answers to simple questions [17,18].

### Exposure assessment

Frequency of alcohol consumption was recorded as 'ex-drinker', 'never', 'less than 1 day monthly', '1–3 days monthly', '1–3 days weekly' and 'at least four days weekly'. Amount ('about 1/4 cup', 'about 1/2 cup', 'about 1 cup', '2–3 cups', '4 cups or above') and type of alcohol ('beer', 'western table wine/grape wine', 'spirit', 'Chinese wine', 'medicinal wine', 'various wines') were also recorded and used to estimate the average intake in grams of ethanol per occasion. As a reliability check, repeat interviews were conducted by telephone on a random sample of 235 decedents and 106 living controls about 3 weeks after the initial interview. The percentage agreement on drinking frequency was 66% in decedents and 73% in living controls.

Alcohol use was categorized as ex-drinker, never, occasional (less than once a week), moderate (at least weekly and per occasion less than or equal to 27.4 gram ethanol (men) or 13.7 gram ethanol (women)) and high (at least weekly and per occasion more than 27.4 gram ethanol (men) or 13.7 gram ethanol (women)). This categorization avoids any potential bias from grouping 'sick quitters' with never-drinkers, identifies occasional drinkers and reflects US Dietary Guidelines for Americans 2005 [19] on 'drinking in moderation', i.e. no more than 2 standard drinks per day in men and 1 standard drink per day in women, where a standard drink is 13.7 gram ethanol. Moreover binge drinking is rare in Chinese [20], so it is unlikely that moderate, but regular, drinkers were on average drinking more than those classified as high alcohol users. Causes of death were obtained from the official registrar and classified according to the World Health Organisation's ninth international classification of diseases (ICD-9) by the Department of Health, Hong Kong Government. Almost all deaths in Hong Kong are certified by hospital doctors and validity and completeness of the causes of death should be good; Hong Kong cause of death coding has been used in other studies and found to be of good quality [16].

### Analytic strategies

We used two different analytic strategies. First, we used living controls. Second, we used as controls all deaths from non-alcohol related causes, i.e. excluding deaths from all respiratory or vascular diseases, i.e. including stroke, (ICD-9 390–519, 11, 18), alcohol related cancers (lip, oropharyngeal, esophageal, liver, laryngeal and breast, ICD-9 140-1, 143–146, 148–50, 155, 161, 174) and liver cirrhosis (ICD-9 571) [15]. Consistent with the selection of living controls and to ensure an adequate number similar to the general population in socio-economic status, we restricted all analyses to those aged 60 years or over.

### Outcomes

The major outcomes were cause-specific mortality from IHD (ICD-9 410–414) and COPD (ICD-9 416-7, 491-2, 496). Stroke was not considered as an outcome because strokes are difficult to classify after death, and were mainly recorded as unknown (62%) with 31% hemorrhagic and 7% ischemic. A secondary outcome for content validity was alcohol related cancers and liver cirrhosis.

### Statistical analysis

Unconditional logistic regression adjusted for potential confounders was used to assess the association of alcohol use with mortality in each analytic strategy. Whether the association of alcohol use with mortality varied with sex and/or smoking status was assessed by comparing model fit, using the Akaike Information Criterion, for the 9 different models with all possible combinations of 2-way and 3-way interactions between alcohol use, sex and smoking status. Potential confounding factors were age (in 5 year age-bands) and leisure-time exercise, education, physical activity in longest held job, smoking status and place of birth categorized as in Table 1. Apart from age, education and smoking these were included in the final model on a change in estimate criteria for the association of alcohol use with IHD mortality using living controls, on which basis place of birth was dropped. Complete data were available for 94% of the observations; missing data were excluded; p values of less than 0.05 were taken to indicate statistical significance. The project received ethics approval from the Ethics Committee of the Faculty of Medicine, University of Hong Kong.

**Table 1:** Characteristics of cases and controls for Chinese men and women aged 60 years and over from Hong Kong in 1998

		Men				Women			
		Cases		Controls		Cases		Controls	
		Cause of death				Cause of death			
Characteristic		IHD	COPD	Living	Dead – Non-vascular, non-respiratory and non-alcohol related deaths*	IHD	COPD	Living	Dead – Non-vascular, non-respiratory and non-alcohol related deaths*

	N	1174	985	3274	4879	1096	456	7046	4164
Age	Mean	75.6	76.5	73.6	73.9	80.2	80.6	71.0	77.7
	(standard error)	(0.24)	(0.25)	(0.14)	(0.12)	(0.26)	(0.37)	(0.09)	(0.14)
									
Education	No formal (%)	25	33	26	28	69	70	57	65
	Primary (%)	49	49	49	48	22	24	33	26
	Secondary (%)	26	18	24	24	9	6	10	9
									
Job type	Non-economically active (%)	1	0	1	0	48	48	46	43
	Sedentary (%)	20	14	17	16	5	5	5	6
	Light (%)	23	18	20	18	23	18	20	22
	Moderate (%)	40	44	41	43	17	20	19	21
	Heavy (%)	16	23	21	22	7	8	9	9
									
Birthplace	Hong Kong (%)	14	9	11	12	12	11	12	13
									
*10 years previously*									
Exercise	None or <1/month (%)	65	76	57	68	64	73	55	65
									
Smoking	Never (%)	37	14	39	29	79	40	86	77
	Amount unknown (%)	3	4	3	3	2	4	0	1
	<1/day (%)	2	2	1	1	2	2	1	1
	1–14/day (%)	27	29	29	26	12	30	9	13
	15–24/day (%)	21	36	23	31	4	18	3	6
	25+/day (%)	9	16	6	11	1	7	1	2
									
Alcohol use	Never (%)	57	52	47	46	88	82	88	88
	Occasional (%)	11	10	16	13	6	7	6	6
	Moderate (%)	12	14	21	17	2	3	2	2
	High (%)	7	10	8	12	1	2	2	2
	Ex-drinker (%)	13	13	8	12	3	6	1	3
									
Mean alcohol per occasion (grams of ethanol)									
	Occasional	19.6	22.5	19.0	21.1	16.4	21.0	13.7	17.4
	(standard error)	(2.44)	(3.13)	(1.12)	(1.35)	(2.21)	(3.64)	(0.62)	(1.51)
	Moderate	15.6	17.1	15.5	16.0	7.4	6.0	7.6	7.8
	(standard error)	(0.70)	(0.69)	(0.31)	(0.29)	(0.75)	(0.16)	(0.21)	(0.32)
	High	118.7	116.7	108.1	106.1	30.8	48.1	50.2	54.8
	(standard error)	(9.39)	(7.99)	(4.92)	(3.18)	(4.74)	(9.30)	(4.92)	(7.01)
	Ex-drinker	38.8	48.5	36.1	44.5	16.9	39.4	20.8	24.9
	(standard error)	(4.21)	(6.08)	(2.79)	(2.82)	(3.76)	(11.3)	(3.46)	(4.10)
									
Main type of alcohol in current-drinkers (n)		349	338	1456	2026	93	53	718	390
	Beer (%)	30	18	29	21	15	4	19	12
	Western table/grape wine (%)	2	1	3	2	8	4	3	4
	Sprits (%)	21	19	18	18	20	34	21	21
	Chinese wine (%)	16	27	15	20	10	11	9	15
	Medicinal wine (%)	7	6	5	4	26	21	22	22
	Various wines (%)	24	30	30	34	22	26	25	26

IHD = ischemic heart disease; COPD = chronic obstructive pulmonary disease

* excludes deaths coded as ICD-9 11,18, 140, 141, 143–146, 148, 149, 150, 155, 161, 174, 390–519, 571

### Validation

Consistent with the carcinogenic and liver damaging effects of alcohol, high alcohol use and ex-drinking were associated with higher mortality from alcohol related disease (cancers or liver cirrhosis) when using living controls [see Additional file 1]. Moreover, there was a clear, biologically plausible, monotonically increasing risk with increasing alcohol use when using dead controls, which was not evident when using living controls; thus demonstrating a difference between the two analytic strategies.

## Results

Table 1 shows the characteristics of the cases and the two sets of controls (living and dead). There were 1174 male and 1096 female IHD cases and 985 male and 456 female COPD cases. There were 3274 male and 7046 female living controls, and 4879 male and 4164 female dead controls. About half the men and 13% of the women were reported as ever using alcohol. Occasional use was most common in women; men were more evenly split between the occasional, moderate, high and ex-drinking groups. Alcohol intakes were relatively low, especially in women; for the high women users average intake per occasion was less than 55 grams of ethanol. Western table wine or grape wine was consistently the least frequently reported usual type of alcohol. For both the cases and controls, older people were more likely to be ex-drinkers (data not shown).

Using living controls there was some evidence that the association of alcohol use with IHD mortality differed in men and women [see additional file 2]. In men any current alcohol use, occasional, moderate or high, was associated with lower IHD mortality (Table 2). In women occasional alcohol use was unrelated to IHD mortality, but high and moderate use were associated with lower IHD mortality. In contrast, using non-alcohol related deaths as controls, alcohol use had different associations with death from IHD by smoking status, but similar associations in men and women [see additional file 2]. In never-smokers, occasional and moderate alcohol use was not associated with death from IHD, with odds ratios slightly above unity. However, high alcohol use was negatively associated with death from IHD in never-smokers. In ever-smokers, occasional, moderate and high alcohol use were all negatively associated with death from IHD. Ex-drinking was positively associated with IHD death using living controls, but not using non-alcohol related deaths as controls.

**Table 2:** Adjusted† associations of alcohol use with death from ischemic heart disease (ICD-9 410–414) in different analytic strategies for Chinese men and women aged 60 years and over from Hong Kong in 1998

			Alcohol use, 10 years previously								
			Never	Occasional		Moderate		High		Ex-drinker	
Analytic strategy	Control group	Stratification		<1/week		At least weekly, and ≤ 13.7 g (women) or ≤ 27.4 g (men) of ethanol/occasion		At least weekly, and >13.7 g (women) or >27.4 g (men) of ethanol/occasion			
				OR	95% CI	OR	95% CI	OR	95% CI	OR	95% CI
Living control	All living controls	Men	1	0.58	0.47 to 0.72	0.51	0.41 to 0.62	0.63	0.47 to 0.84	1.30	1.03 to 1.64
		#cases/controls	667/1552		132/521		144/688		77/256		154/257
		Women	1	1.04	0.77 to 1.40	0.58	0.34 to 1.00	0.43	0.20 to 0.89	1.82	1.16 to 2.84
		#cases/controls	968/6222		67/454		18/160		9/113		34/97
		Ever-smoker	1	0.64	0.51 to 0.81	0.50	0.39 to 0.63	0.63	0.47 to 0.83	1.35	1.06 to 1.71
		#cases/controls	502/1398		124/509		114/571		75/276		149/251
		Never-smoker	1	0.84	0.64 to 1.11	0.73	0.52 to 1.04	0.58	0.30 to 1.14	1.47	0.97 to 2.23
		#cases controls	1133/6376		75/466		48/277		11/92		39/103
											
Dead control*	All non-vascular, non-respiratory deaths and non-alcohol related deaths*	Men	1	0.75	0.61 to 0.93	0.66	0.54 to 0.81	0.56	0.43 to 0.73	0.95	0.77 to 1.17
		#cases/controls	667/2249		132/623		144/841		77/581		154/585
		Women	1	1.14	0.85 to 1.52	0.93	0.55 to 1.57	0.48	0.24 to 0.97	1.01	0.68 to 1.50
		#cases/controls	968/3650		67/236		18/82		9/74		34/122
		Ever-smoker	1	0.75	0.60 to 0.93	0.58	0.46 to 0.73	0.53	0.41 to 0.69	0.89	0.72 to 1.10
		#cases/controls	502/1890		124/604		114/761		75/583		149/600
		Never-smoker	1	1.05	0.80 to 1.38	1.07	0.76 to 1.50	0.51	0.27 to 0.98	1.17	0.80 to 1.71
		#cases/controls	1133/4009		75/255		48/162		11/72		39/107

†All models adjusted for age, sex, education, physical activity, physical activity in longest held occupation and smoking, except in cases where stratified results are presented for a co-variable, or co-variables.

* excludes deaths coded as ICD-9 11, 18, 140, 141, 143–146, 148, 149, 150, 155, 161, 174, 390–519, 571

Occasional, moderate and high alcohol use were generally associated with lower COPD mortality in both analytic strategies (Table 3), although the effect of occasional and moderate drinking was smaller when using dead controls. There was some evidence that the association of alcohol use with COPD mortality differed in men and women [see additional file 2], such that occasional and moderate alcohol use was not significantly associated with lower COPD mortality in women when using non-alcohol related deaths as controls.

**Table 3:** Adjusted† associations of alcohol use with mortality from COPD (ICD-9 416-7, 491-2, 496) in different analytic strategies for Chinese men and women aged 60 years and over from Hong Kong in 1998

			Alcohol use, 10 years previously								
			
			Never	Occasional		Moderate		High		Ex-drinker	
Analytic strategy	Control group	Stratification		<1/week		At least weekly, and ≤ 13.7 g (women) or ≤ 27.4 g (men) of ethanol/occasion		At least weekly, and >13.7 g (women) or >27.4 g (men) of ethanol/occasion			
				OR	95% CI	OR	95% CI	OR	95% CI	OR	95% CI
Living control	All living controls	Men	1	0.48	0.38 to 0.62	0.46	0.37 to 0.57	0.66	0.50 to 0.86	0.92	0.72 to 1.19
		#cases/controls	514/1552		99/521		139/688		102/256		131/257
		Women	1	0.64	0.41 to 0.99	0.74	0.39 to 1.40	0.49	0.23 to 1.04	2.08	1.22 to 3.56
		#cases/controls	374/6222		30/454		14/160		10/113		28/97
		Ever-smoker	1	0.57	0.46 to 0.72	0.52	0.41 to 0.65	0.66	0.51 to 0.86	1.08	0.84 to 1.38
		#cases/controls	602/1398		121/509		143/571		107/276		151/251
		Never-smoker	1	0.27	0.13 to 0.57	0.43	0.22 to 0.86	1.03	0.40 to 2.66	0.96	0.44 to 2.09
		#cases/controls	286/6376		8/466		10/277		5/92		8/103
											
Dead control *	All non-vascular to non-respiratory deaths and non-alcohol related deaths*	Men	1	0.59	0.46 to 0.75	0.58	0.47 to 0.72	0.58	0.46 to 0.74	0.67	0.53 to 0.83
		#cases/controls	514/2249		99/623		139/841		102/581		131/585
		Women	1	0.79	0.52 to 1.20	0.88	0.48 to 1.61	0.63	0.31 to 1.25	1.05	0.67 to 1.65
		#cases/controls	374/3650		30/236		14/82		10/74		28/122
		Ever-smoker	1	0.67	0.54 to 0.84	0.62	0.51 to 0.77	0.60	0.47 to 0.75	0.73	0.59 to 0.90
		#cases/controls	602/1890		121/604		143/761		107/583		151/600
		Never-smoker	1	0.38	0.18 to 0.78	0.67	0.34 to 1.30	0.68	0.27 to 1.72	0.74	0.35 to 1.56
		#cases/controls	286/4009		8/255		10/162		5/72		8/107

†All models were adjusted for age, sex, education, physical activity, physical activity in longest held occupation and smoking, except in cases where stratified results are presented for a co-variable or co-variables.

* excludes deaths coded as ICD-9 11, 18, 140, 141, 143–146, 148, 149, 150, 155, 161, 174, 390–519, 571

## Discussion

In a large population-based Chinese sample using two different analytic strategies, we found consistent with many other studies [1], that reasonably high alcohol use was associated with lower IHD mortality, and also that high alcohol use was usually associated with lower COPD mortality. This same high level of alcohol use was also associated with higher mortality from alcohol related diseases. On the other hand, the protective effect of occasional and moderate alcohol use was reduced using an analytic strategy potentially less open to residual confounding and other biases, such that occasional and moderate alcohol use only appeared to be specifically protective for IHD in ever-smokers and for COPD in men.

That reasonably high levels of alcohol use were associated with lower IHD mortality is biologically plausible, through increasing HDL-cholesterol [21], although alcohol use may raise blood pressure [22]. The physiological reason for the association between high alcohol use and lower COPD mortality is not clear, but could be due to associated protection from underlying cardiovascular disease [3] or anti-inflammatory effects [23]. Because of the generally restrained pattern of alcohol use in Chinese [20], high alcohol use defined as more than 'drinking in moderation' may represent levels of use (30–40 g ethanol per day) commonly accepted as normal, moderate and protective in other populations. Such a downward shift in the distribution of alcohol use would explain the large protective effect of high alcohol use, because our high alcohol users may imbibe sufficient to improve lipid profiles without consuming enough to induce cardiomyopathy. However, these same levels of alcohol use were also positively associated with death from alcohol related cancers/liver cirrhosis.

Occasional and moderate alcohol use had somewhat inconsistent associations with IHD and COPD mortality by sex and analytic strategy, with perhaps a less protective effect using a strategy less open to residual confounding and other biases, illustrating the difficulty of estimating the impact of low to moderate alcohol use in observational studies. Nevertheless, our findings using living controls are consistent with other studies. Occasional alcohol use has previously been found protective against IHD [8], although the biological plausibility is unclear, and the apparent protection has been suggested as due to residual confounding [8], differential by sex, as would be expected from an activity which has sex-specific cultural and social attributes. Moderate alcohol use has been found protective against ischemic heart disease in Chinese [11,12], as has alcohol use against COPD mortality in Chinese men [24]. On the other hand, using the dead control analytic strategy we found that moderate alcohol use did not protect against IHD in never-smokers, although it was associated with lower mortality from COPD in men. Few studies have formally examined whether alcohol use has a different effect on IHD mortality by smoking status, with mixed results [25,26]. Nevertheless, our findings are similar to the observation that moderate alcohol use was not associated with a lower risk of IHD in people with healthy behavior including non-smoking, but was associated with a lower risk in people with less healthy behavior [27]. No studies have examined whether alcohol use has a different effect on COPD by sex. However, the apparent protective effect of alcohol against COPD in Chinese men could be due to factors other than the action of alcohol. In Chinese there is a common genetic polymorphism of the aldehyde dehydrogenase gene (ALDH2*2) which makes alcohol use a less pleasant experience, due to the build up of acetaldehyde. Acetaldehyde is ubiquitous [28] and associated with bronchoconstriction [29]. Chinese women rarely use alcohol. Never drinking in men may correspond most closely to possession of ALDH2*2 alleles, making male never-users more vulnerable to acetaldehyde, and associated bronchoconstriction, thereby generating an apparent protective effect of alcohol in male ever-users. Given these considerations and the rising use of alcohol in China, better designed studies, such as using Mendelian randomization techniques, are urgently needed to clarify the role of low to moderate alcohol use in IHD and COPD.

### Strengths and limitations

The findings from this study come from a large population based case-control study, which enabled us to cross check our findings using different analytic strategies, to focus on the specificity of the alcohol effects and largely remove the effect of alcohol as a marker of better health status and other health related factors. We were able to adjust for socio-economic status, using two indicators, and lifestyle. More importantly the size of the study made it possible to examine differences by disease and within some sub-groups, although there were few never-smokers who died of COPD, and few women who drank large amounts. Nevertheless, there are limitations. First, the setting only allowed crude alcohol use assessment, obtained from a third-party with all the caveats that entails. However satisfactory repeatability was attained for alcohol use, and alcohol use was associated with an increased risk of death from alcohol related diseases. Second, this study is subject to recall bias and the possible ascribing of unhealthy drinking habits to the dead; however this would not affect the analytic strategy using dead controls. There is also little reason to suspect such a strategic bias because the main focus was to assess the effects smoking on mortality. Conversely, a downward bias in the reporting of alcohol use is possible, particularly for women, where alcohol use is rare. However the pattern of proxy-reported alcohol use is comparable with the pattern of self-reported alcohol use in other studies of older people in Hong Kong [13]. Third, our study is in older people from a social and cultural setting where alcohol use is typically light and where there are many never-drinkers, especially amongst never smokers and amongst women. Therefore, we did not have sufficient power to detect very small protective effects of alcohol use. Nevertheless, despite low numbers, we found that excessive alcohol use was consistently negatively associated with death from IHD. We were also able to find evidence that the association of alcohol use with death from IHD varied with ever-smoking status, although we cannot rule out the possibility that this was a chance finding. Fourth, our study from a setting where red grape wine is rarely used means we cannot comment on moderate use of red wine. Nevertheless these limitations do not detract from our observations comparing the associations with alcohol using different analytic strategies, or our findings concerning alcohol. Fifth, the living controls were selected from the same base population as the cases, and were usually the spouse (78%) or other family member (21%) of the case. As such they are a selection of people similar to the cases, who were unlikely to have been selected on the basis of their alcohol use. It was not feasible to collect detailed health status in this study, so it is possible that the living controls differed from the cases on health status 10 years previously, which would cause biases if there is differential relative dose response to alcohol use (i.e. effect modification) by underlying health status or if the cases had already changed their alcohol use pattern because of ill-health (reverse causality). Hence our presentation of results using dead controls, whose health status 10 years previously may have been more similar to the cases. Finally, our findings may not be generalizable beyond Chinese people, because of the different patterns of alcohol use in Chinese society and the different distribution of alcohol processing genes. However, Chinese people do constitute a substantial proportion of the global population. Moreover, our findings do have similarities with findings elsewhere, including the finding that moderate alcohol use is not protective against IHD in non-smokers with healthy behavior [27].

## Conclusion

Reasonably high levels of alcohol use in an older Chinese population were associated with lower IHD and possibly COPD mortality. Given the generally low levels of alcohol use in China, these levels although higher than 'drinking in moderation' might correspond to levels accepted as moderate elsewhere. Nevertheless, these same levels of alcohol use were also positively associated with mortality from alcohol related cancers and liver cirrhosis. Moderate alcohol use was less consistently protective against IHD mortality, whilst occasional and moderate alcohol use were associated with lower COPD mortality particularly in men, either due to some yet to be clarified properties of alcohol or as the artefactual result of genetic selection into alcohol use in a Chinese population. Given the increasing use of alcohol in China with economic development, other designs and analytic strategies are needed to assess the impact of alcohol in this population, so that a public health policy based on appropriate evidence can be formulated.

## Competing interests

The authors declare that they have no competing interests.

## Authors' contributions

CMS and GML designed this analysis and drafted the manuscript. THL, SYH and KHM designed the original study and acquired the data. YH carried out the initial data analysis. All authors revised the manuscript critically for important intellectual content; and gave final approval of the version to be published.

## Pre-publication history

The pre-publication history for this paper can be accessed here:



## Supplementary Material

Additional file 1 **Adjusted† associations of alcohol use with death from alcohol related cancers and liver cirrhosis‡ using different analytic strategies for Chinese men and women aged 60 years and over from Hong Kong in 1998.** The data provided represent the statistical analysis of the association of alcohol use with death from alcohol related cancers. Click here for file

Additional file 2 **Akaike Information Criterion statistics for adjusted† models using both analytic strategies containing all the 9 different possible combinations of interactions between sex, smoking and alcohol use.** The data provide an assessment of model fit. Click here for file

## References

[B1] Corrao G, Rubbiati L, Bagnardi V, Zambon A, Poikolainen K (2000). Alcohol and coronary heart disease: a meta-analysis. Addiction.

[B2] Reynolds K, Lewis B, Nolen JD, Kinney GL, Sathya B, He J (2003). Alcohol consumption and risk of stroke: a meta-analysis. JAMA.

[B3] Doll R, Peto R, Boreham J, Sutherland I (2005). Mortality in relation to alcohol consumption: a prospective study among male British doctors. Int J Epidemiol.

[B4] Hart CL, Smith GD, Hole DJ, Hawthorne VM (1999). Alcohol consumption and mortality from all causes, coronary heart disease, and stroke: results from a prospective cohort study of scottish men with 21 years of follow up. BMJ.

[B5] Strandberg AY, Strandberg TE, Salomaa VV, Pitkala K, Miettinen TA (2004). Alcohol consumption, 29-y total mortality, and quality of life in men in old age. Am J Clin Nutr.

[B6] Fillmore KM, Golding JM, Graves KL, Kniep S, Leino EV, Romelsjo A (1998). Alcohol consumption and mortality. I. Characteristics of drinking groups. Addiction.

[B7] Naimi TS, Brown DW, Brewer RD, Giles WH, Mensah G, Serdula MK (2005). Cardiovascular risk factors and confounders among nondrinking and moderate-drinking U.S. adults. Am J Prev Med.

[B8] Fuchs FD, Chambless LE, Folsom AR, Eigenbrodt ML, Duncan BB, Gilbert A (2004). Association between alcoholic beverage consumption and incidence of coronary heart disease in whites and blacks: the Atherosclerosis Risk in Communities Study. Am J Epidemiol.

[B9] Fillmore KM, Kerr WC, Stockwell T, Chikritzhs T, Bostrom A (2006). Moderate alcohol use and reduced mortality risk: systematic error in prospective studies. Addiction Research and Theory.

[B10] Lawlor DA, Smith GD, Ebrahim S (2004). Commentary: the hormone replacement-coronary heart disease conundrum: is this the death of observational epidemiology?. Int J Epidemiol.

[B11] Yuan JM, Ross RK, Gao YT, Henderson BE, Yu MC (1997). Follow up study of moderate alcohol intake and mortality among middle aged men in Shanghai, China. BMJ.

[B12] Xu WH, Zhang XL, Gao YT, Xiang YB, Gao LF, Zheng W (2007). Joint effect of cigarette smoking and alcohol consumption on mortality. Prev Med.

[B13] Schooling CM, Sun W, Ho SY, Chan WM, Tham MK, Ho KS (2008). Moderate alcohol use and mortality from ischaemic heart disease: a prospective study in older Chinese people. PLoS ONE.

[B14] Calle EE (1984). Criteria for selection of decedent versus living controls in a mortality case-control study. Am J Epidemiol.

[B15] Rehm J, Room R, Graham K, Monteiro M, Gmel G, Sempos CT (2003). The relationship of average volume of alcohol consumption and patterns of drinking to burden of disease: an overview. Addiction.

[B16] Lam TH, Ho SY, Hedley AJ, Mak KH, Peto R (2001). Mortality and smoking in Hong Kong: case-control study of all adult deaths in 1998. BMJ.

[B17] Herrmann N (1985). Retrospective information from questionnaires. I. Comparability of primary respondents and their next-of-kin. Am J Epidemiol.

[B18] Lerchen ML, Samet JM (1986). An assessment of the validity of questionnaire responses provided by a surviving spouse. Am J Epidemiol.

[B19] CDC (2006). Dietary Guidelines for Americans. web. http://www.cdc.gov/.

[B20] Janghorbani M, Ho SY, Lam TH, Janus ED (2003). Prevalence and correlates of alcohol use: a population-based study in Hong Kong. Addiction.

[B21] Hines LM (2004). Genetic modification of the effect of alcohol consumption on CHD. Proc Nutr Soc.

[B22] Chen L, Smith GD, Harbord RM, Lewis SJ (2008). Alcohol intake and blood pressure: a systematic review implementing a Mendelian randomization approach. PLoS Med.

[B23] Sisson JH, Stoner JA, Romberger DJ, Spurzem JR, Wyatt TA, Owens-Ream J (2005). Alcohol intake is associated with altered pulmonary function. Alcohol.

[B24] Reilly KH, Gu D, Duan X, Wu X, Chen CS, Huang J (2008). Risk factors for chronic obstructive pulmonary disease mortality in Chinese adults. Am J Epidemiol.

[B25] Ebbert JO, Janney CA, Sellers TA, Folsom AR, Cerhan JR (2005). The association of alcohol consumption with coronary heart disease mortality and cancer incidence varies by smoking history. J Gen Intern Med.

[B26] Klatsky AL, Armstrong MA, Friedman GD (1990). Risk of cardiovascular mortality in alcohol drinkers, ex-drinkers and nondrinkers. Am J Cardiol.

[B27] Britton A, Marmot MG, Shipley M (2008). Who benefits most from the cardioprotective properties of alcohol consumption – health freaks or couch potatoes?. J Epidemiol Community Health.

[B28] Miyake T, Shibamoto T (1993). Quantitative Analysis of Acetaldehyde in Foods and Beverages. J Agric Food Chem.

[B29] Kawano T, Matsuse H, Kondo Y, Machida I, Saeki S, Tomari S (2004). Acetaldehyde induces histamine release from human airway mast cells to cause bronchoconstriction. Int Arch Allergy Immunol.

