# Supplementary material for: Alcohol and cardio-respiratory deaths in Chinese: a population-based case-control study of 32,462 older Hong Kong adults
Source: BMC Public Health. 2009 Feb 5;9:49. doi: 10.1186/1471-2458-9-49 (PMC2649071; doi:10.1186/1471-2458-9-49)
Supplement: Additional file 1 — Adjusted† associations of alcohol use with death from alcohol related cancers and liver cirrhosis‡ using different analytic strategies for Chinese men and women aged 60 years and over from Hong Kong in 1998. The data provided represent the statistical analysis of the association of alcohol use with death from alcohol related cancers. [file 1471-2458-9-49-S1.doc]

Adjusted† associations of alcohol use with death from alcohol related cancers and liver cirrhosis‡ using different analytic strategies for Chinese men and women aged 60 years and over from Hong Kong in 1998

|  |  |  | Alcohol use, 10 years previously | | | | | | | | |
| --- | --- | --- | --- | --- | --- | --- | --- | --- | --- | --- | --- |
|  |  |  | Never | Occasional | | Moderate | | High | | Ex-drinker | |
| Analytic strategy | Control group | Stratification |  | <1/week | | At least weekly, and ≤13.7g (women) or ≤27.4g (men) of ethanol/ occasion | | At least weekly, and >13.7g (women) or >27.4g (men) of ethanol/ occasion | |  | |
|  |  |  |  | OR | 95% CI | OR | 95% CI | OR | 95% CI | OR | 95% CI |
| Living control | All living controls | Men | 1 | 0.95 | 0.74 to 1.20 | 1.09 | 0.88 to 1.34 | 2.91 | 2.29 to 3.69 | 2.49 | 1.93 to 3.20 |
|  |  | Women | 1 | 0.93 | 0.64 to 1.34 | 0.70 | 0.37 to 1.33 | 1.34 | 0.74 to 2.42 | 1.87 | 1.07 to 3.29 |
|  |  | Ever-smoker | 1 | 1.03 | 0.79 to 1.33 | 1.14 | 0.90 to 1.44 | 2.81 | 2.19 to 3.59 | 2.43 | 1.86 to 3.18 |
|  |  | Never-smoker | 1 | 0.84 | 0.60 to 1.16 | 1.05 | 0.74 to 1.49 | 2.31 | 1.44 to 3.70 | 2.26 | 1.46 to 3.48 |
|  |  |  |  |  |  |  |  |  |  |  |  |
| Dead control * | All non-vascular, non-respiratory deaths and non-alcohol related deaths* | Men | 1 | 1.34 | 1.06 to 1.70 | 1.55 | 1.26 to 1.90 | 2.43 | 1.96 to 3.02 | 1.96 | 1.56 to 2.45 |
|  |  | Women | 1 | 1.14 | 0.78 to 1.69 | 1.11 | 0.58 to 2.12 | 1.66 | 0.92 to 3.01 | 1.17 | 0.68 to 2.01 |
|  |  | Ever-smoker | 1 | 1.06 | 0.85 to 1.34 | 1.42 | 1.13 to 1.78 | 2.25 | 1.80 to 2.81 | 1.72 | 1.35 to 2.19 |
|  |  | Never-smoker | 1 | 1.15 | 0.83 to 1.61 | 1.59 | 1.11 to 2.29 | 2.26 | 1.39 to 3.66 | 1.99 | 1.30 to 3.05 |
|  |  |  |  |  |  |  |  |  |  |  |  |
|  |  |  |  |  |  |  |  |  |  |  |  |

†All models adjusted for age, sex, education, physical activity, physical activity in longest held occupation and smoking, except in cases where stratified results are presented for a co-variable, or co-variables.

‡All deaths coded as ICD 9 140, 141, 143-146, 148, 149, 150, 155, 161, 174, 571

*all deaths excluding deaths coded as ICD-9 11, 18, 140, 141, 143-146, 148, 149, 150, 155, 161, 174, 390-519, 571
