# Supplementary material for: Alcohol and cardio-respiratory deaths in Chinese: a population-based case-control study of 32,462 older Hong Kong adults
Source: BMC Public Health. 2009 Feb 5;9:49. doi: 10.1186/1471-2458-9-49 (PMC2649071; doi:10.1186/1471-2458-9-49)
Supplement: Additional file 2 — Akaike Information Criterion statistics for adjusted† models using both analytic strategies containing all the 9 different possible combinations of interactions between sex, smoking and alcohol use. The data provide an assessment of model fit. [file 1471-2458-9-49-S2.doc]

Akaike Information Criterion statistics for adjusted† models using both analytic strategies containing all the 9 different possible combinations of interactions between sex, smoking and alcohol use.

| Outcome | IHD | | COPD | |
| --- | --- | --- | --- | --- |
| Analytic strategy | Living control | Dead control * | Living control | Dead control * |
| Interaction terms in the model |  |  |  |  |
| Main effects only | 10612 | 11172 | 6764 | 7761 |
| Sex by alcohol use | 10608 | 11173 | 6757 | 7759 |
| Alcohol use by ever-smoking | 10614 | 11169 | 6769 | 7766 |
| Sex by ever-smoking | 10610 | 11172 | 6751 | 7749 |
| Sex by alcohol use and sex by ever smoking | 10607 | 11174 | 6748 | 7751 |
| Sex by alcohol use and alcohol use by ever smoking | 10613 | 11173 | 6760 | 7763 |
| Sex by ever-smoking and alcohol use by ever-smoking | 10614 | 11171 | 6754 | 7754 |
| Sex by alcohol use, alcohol use by ever smoking and sex by ever-smoking | 10613 | 11175 | 6750 | 7756 |
| Sex by alcohol use by ever-smoking | 10621 | 11181 | 6754 | 7760 |
|  |  |  |  |  |

†adjusted for age, sex, education, physical activity, physical activity in longest held occupation and smoking

* all deaths excluding deaths coded as ICD-9 11, 18, 140, 141, 143-146, 148, 149, 150, 155, 161, 174, 390-519, 571

A smaller Akaike Information Criterion (AIC) indicates a better fitting model.
